# Assessment of the effectiveness of a polypropylene onlay mesh in the prevention of laparoscopic trocar-site incisional hernia in high-risk patients. A randomized clinical trial

**DOI:** 10.1007/s10029-024-03124-7

**Published:** 2024-08-29

**Authors:** Ana Ciscar, Emma Sánchez-Sáez, Marina Vila Tura, Patricia Ruiz de Leon, Marta Gomez Pallarès, Daniel Troyano Escribano, Marta Abadal Prades, Esther Mans Muntwyler, José-Antonio Pereira, Josep M. Badia

**Affiliations:** 1 Department of Surgery, UD de Medicina de la Vall d’Hebron, Universitat Autonoma de Barcelona, Edifici W, Passeig Vall d’Hebron, Barcelona, 08035 Spain; 2 Hospital de Sant Boi, Parc Sanitari Sant Joan de Deu, Sant Boi, Spain; 3 Hospital Universitari de Bellvitge, Hospitalet del Llobregat, Spain; 4 Fundació Hospital Asil de Granollers, Granollers, Spain; 5 Hospital de Calella, Corporació de Salut del Maresme I La Selva, Calella, Spain; 6 Fundació Hospital de L’Esperit Sant, Santa Coloma de Gramenet, Spain; 7 Hospital de Mataró. Department of Radiology. Consorci Sanitari del Maresme, Mataró, Spain; 8 Hospital de Mataró. Department of Surgery. Consorci Sanitari del Maresme, Mataró, Spain; 9 Abdominal Wall Unit, Department of General Surgery, Hospital del Mar. Parc de Salut Mar, Barcelona, Spain; 10 Hospital de Granollers, Granollers, Spain; 11 School of Medicine, Universitat Internacional de Catalunya, Sarrià-Sant Gervasi, Barcelona Spain; 12 Hospital Universitari Vall d’Hebron, Barcelona, Spain; 13 Medicine and Life Sciences Department, Universitat Pompeu Fabra, Barcelona, Spain

**Keywords:** Trocar site incisional hernia, Port site hernia, Incidence, Prophylactic mesh, Randomized clinical trial

## Abstract

**Purpose:**

Trocar site incisional hernia (TSIH) is a common complication of laparoscopic surgery. In the scientific literature there are few descriptions of methods or tools for its prevention. The aim of this report was to assess the effectiveness and safety of a prophylactic measure designed to lower rates of TSIH.

**Methods:**

A multicenter randomized double-blinded clinical trial was performed in high-risk patients (diabetes mellitus and/or age ≥ 70 years and/or BMI ≥ 30 kg/m^2^ and/or extended incision for specimen retrieval) who underwent either elective or emergency laparoscopic cholecystectomy. Patients were assigned to prophylactic onlay polypropylene mesh fixation (intervention) or to standard trocar closure (control). The main aim was to analyze the efficacy of the intervention, taking occurrence of TSIH as the primary outcome. Clinical and radiological follow up lasted at least one year after surgery. Secondary endpoints were technique-related complications (surgical site occurrences).

**Results:**

One hundred and forty-three patients were randomized and finally 116 were analyzed (64 in the intervention arm and 52 in the control arm). Groups were homogeneous. Mean [SD] age, 65 [18] years; 86 (60.6%) were women. The cumulative TSIH incidence was lower in the intervention group although the differences did not reach statistical significance, assessed either radiologically (16 [25.4%] vs 17 [31.5%], p = 0.538) or clinically (9 [16.1%] vs 9 [20], p = 0.613). No differences in surgical site infection, hematoma or seroma were detected. Mean follow-up was 670 days (range 223–1294).

**Conclusion:**

Our results show that, when properly assessed, the overall TSIH incidence is extremely high. Although polypropylene onlay mesh placement is safe, it does not appear to be effective in reducing the TSIH incidence rate. Radiological evaluation may be more accurate.

**Trial registration:**

ClinicalTrials.org NCT03495557. Date of registration: April 12, 2018

## Background

An incisional hernia (IH) is a defect of the abdominal wall in a postoperative scar region, perceptible or palpable through clinical examination and/or imaging tests [1]. After laparoscopic surgery, IH is further defined either as Trocar Site Incisional Hernia (TSIH) or Port Site Hernia (PSH).

TSIH can be diagnosed through a physical examination, but many authors have raised doubts about the accuracy of the results [2]. The current consensus is that imaging studies such as Computed Tomography (CT) or dynamic abdominal sonography for hernia (DASH) improve the diagnostic accuracy of TSIH [3, 4]

The incidences of TSIH reported in recent research do not accurately report the true value [5] for several reasons: they are based on retrospective studies [6], insufficient follow up [7], heterogeneous diagnostic methods, or the absence of systematic physical examination and radiological tests [8–14]. The most reliable recent results suggest an incidence of around 25% [15, 16].

It is clear that a reintervention (either elective or emergency) always involves risk for the patient and incurs considerable expense (up to $15,000) [17] Often, however, the importance of TSIH has been overlooked.

Given the high number of laparoscopic procedures performed annually and the doubts regarding the current rate of complications and the best preventive measures, TSIH has become a major public health problem. To avoid its occurrence, certain authors have proposed the use of a prophylactic mesh in laparoscopic surgery [18–20].

We hypothesized that the placement of a mesh at the umbilical port site in patients undergoing cholecystectomy could lower the rate of TSIH under 23% while not increasing surgical site complications. The main objective of the present study was to evaluate the efficacy of the insertion of an onlay mesh designed to reduce the TSIH rate. Secondary aims were to assess the safety throughout the surgical site occurrences analysis.

## Methods

### Study design

A prospective two-arm (1:1) randomized trial was carried out from January 2018 to April 2021. Patients who had undergone emergency or elective laparoscopic cholecystectomy at five general surgery units in Catalonia, Spain were included. The study was designed to be double-blinded for the patients, who did not know into which group they had been assigned, and for the surgeon and radiologist performing the control visits, who also did not know into which group each patient had been classified. The surgeons performing the intervention knew into which group the patient had been classified, but none of them participated in the clinical evaluation of the study.

The study was approved by the ethics committee of the promoter center, the Consorci Sanitari del Maresme, Mataró, on April 26, 2017 (CEIC 07/17) and it was registered at Clinicaltrials.gov on April 12, 2018, under registration number: NCT 03495557.

### Patients

Patients were included if they met the following criteria: age ≥ 18 years, presence of at least one of the following risk factors for TSIH (age ≥ 70 years, body mass index (BMI) ≥ 30 kg/m^2^, diabetes mellitus (DM) or enlargement of the trocar-related umbilical incision to remove the specimen) and provision of signed written informed consent. Patients who required conversion to subcostal laparotomy, underwent emergency repeat surgery, were immunosuppressed, or were diagnosed with intra- or preoperative umbilical hernia were excluded.

### Procedures

Both groups underwent a laparoscopic cholecystectomy in the French position (where patient lies supine with his legs opened while the surgeon stands in-between the legs and assistants stand on either side of the patient). The pneumoperitoneum was created using Hasson's open technique. The 12 mm umbilical trocar was inserted through a supraumbilical middle line incision, in the fascia one cm above the umbilical root (without disinserting it) at a 90º angle and in a transverse position. The other trocars inserted were another 12 mm trocar in left pararectal position, and two 5 mm trocars, one in the epigastrium and the other in the right hypochondrium. After removal of the specimen (with an endobag), the aponeurosis was closed with an interrupted suture using MonoPlus^®^ polydioxanone absorbable monofilament 2/0 with a 26 mm circular needle (B. Braun, Melsungen, Germany). The stitches were placed at intervals of approximately 4 mm. Hasson’s trocar incision was approximately 12 mm long, and stitches were placed at each end of the incision (to fix the trocar) and another in the center of the incision.

In the intervention group a macropore (Amid’s classification > 75 microm), low molecular weight (USP Class VI) mesh (MallaNets, Llinars del Vallès, Spain) was fixed in the onlay position after the primary closure of the muscular fascia. The mesh, 50 × 52 mm in size, was shaped to adequately cover (minimum 1 cm each edge) the transverse incision which was made 1 cm above the umbilical root in the linea alba. The mesh was fixed using MonoPlus^®^: polydioxanone absorbable monofilament 2/0 with a 26 mm circular needle (B. Braun, Melsungen, Germany) with interrupted stitches arranged in a crown pattern. A standardized postoperative follow-up was carried out, comprising a physical examination performed by a general surgeon at 1, 6 and 12 months, and an abdominal ultrasound performed by a specialist radiologist at 12 months. The ultrasound diagnosis (TSIHu) was taken as the reference diagnosis, so the overall incidence of hernia in the study is the same as TSIHu.

### Measurements and variables

The primary endpoint was defined as the incidence of clinically and radiologically documented TSIH. Secondary variables were the occurrence of seroma, hematoma, and infection at the surgical site. The estimate of expected TSIH cases in the control group was based on a previous retrospective study of our group [21], and TSIH in the control group was estimated based on the probability of umbilical hernia in the general population [22]. Sample size was calculated using the ARCSINUS approach, accepting an alpha risk of 0.05 and a beta risk of less than 0.2 in a bilateral contrast. Seventy subjects were required in each group (group 1, control, and group 2, intervention) to detect statistically significant differences. These differences were expected to be 0.23 for group 1 and 0.05 for group 2. A loss to follow-up rate of 25%.was estimated.

The randomization process was carried out using the simple sealed-envelope method. The surgeon was aware of the group allocation at the time of umbilical trocar closure. The principal investigator provided the randomization envelopes to the coordinators of each center.

### Statistical analysis

Data were obtained from the computerized hospital medical record, clinical interviews, and physical and radiological examinations. Continuous variables were described as means and standard deviations, and categorical variables as absolute numbers and percentages. The Chi-square test was used to compare categorical variables (Fisher’s exact test was used when needed), and the Student t-test for continuous variables. Adjusted odds ratios (OR) were calculated using logistic regression*.* OR with 95% confidence intervals (CI) were presented for each variable studied. Differences were significant at the 5% level. All reported p values were two-sided*.* The Jamovi project (2021) platform was used for the statistical analysis (jamovi version 1.6) computer software: retrieved from https://www.jamovi.org. Sydney, Australia.

## Results

**Fig. 1:**
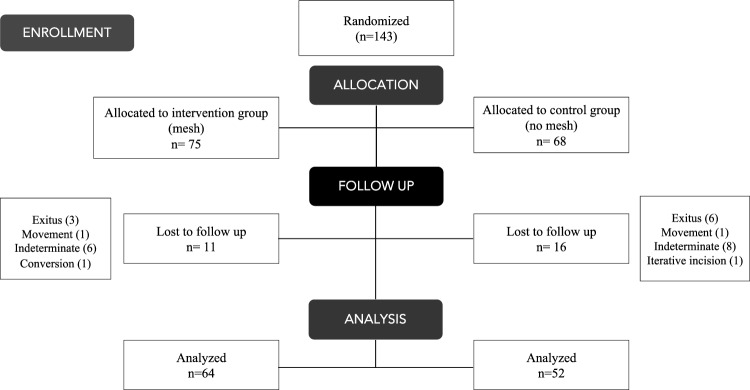
Flow diagram

A total of 143 patients were randomized, 75 to the intervention group (mesh group) and 68 to the control group (no mesh group) Twenty-seven patients (18.9%) were lost to follow up. Finally, 116 patients were analyzed (64 in the intervention arm and 52 in the control arm) (Fig. 1).

Data were analyzed both by intention-to-treat and per protocol. Since the results were similar, the accompanying text and tables show the results by intention-to-treat.

Patients were enrolled from January 2018 to April 2021. Clinical and radiological examination were performed over a 12-month period. Mean postoperative ultrasound follow up was 670 days (almost two years).

Women accounted for 60.6% of the sample, 9.2% were smokers, mean age was 65 years and mean BMI 30.7 kg/m^2^. Over half (54.2%) were ≥ 70 years, 59.6% had a BMI ≥ 30 kg/m^2^, 24.6% DM and 22.7% required a wound enlargement for specimen retrieval. Patients presented a mean of 1.61 risk factors (Table 1), and this number was similar in patients with and without a radiologically diagnosed TSIH (Table 2). The prevalences of ultrasound- and clinically detected TSIH in the overall cohort were 28.2 and 17.8% respectively (Table 1).

**Table 1:** Main patient characteristics and group comparisons

		Total	Mesh	No mesh	p
Main patient characteristics					
N (%)		143	75 (52.4)	68 (47.5)	
Age	Years (SD)	65 (18)	63.5 (19.9)	66.7 (15.4)	
	≥ 70 n (%)	77 (54.2)	41 (55.4)	36 (52.9)	0.635^¶^
	< 70	65 (45.8)	33 (44.6)	32 (47.1)	0.866^ƒ^
Gender n (%)	Male	56 (39.4)	23 (31.1)	33 (48.5)	**0.04**^ƒ^
	Female	86 (60.6)	51 (68.9)	35 (51.5)	
DM n (%)	Yes	35 (24.6)	18 (24.3)	17 (25)	1^ƒ^
	No	107 (75.4)	56 (75.7)	51 (75)	
COPD n (%)	Yes	24 (17.1)	11 (15.3)	13 (19.1)	0.655^ƒ^
	No	116 (82.9)	61 (84.7)	55 (80.9)	
Smokers n (%)	Yes	13 (9.2)	11(15.1)	2 (2.9)	**0.018**^ƒ^
	No	128 (90.8)	62 (84.9)	66 (97.1)	
BMI kg/m^2^ (SD)		30.7 (5.31)	30.9 (5.68)	30.5 (4.92)	0.732^¶^
	≥ 30 n (%)	84 (59.6)	46 (63)	38 (55.9)	0.397^ƒ^
	< 30	57 (40.4)	27 (37)	30 (44.1)	
Previous abdominal surgery n (%)	Yes	47 (33.1)	27 (36.5)	20 (29.4)	0.384^ƒ^
	No	95 (66.9)	47 (63.5)	48 (70.6)	
Total number of risk factors^a^	m (SD)	1.61 (0.7)	1.62 (0.66)	1.60 (0.75)	0.910^¶^
Wound enlargement (%)	Yes	32 (22.7)	14 (19.2)	18 (26.5)	0.322^ƒ^
	No	109 (77.3)	59 (80.8)	50 (73.5)	
Operation time	Minutes (SD)	77 (36.7)	77.7 (35.3)	76.2 (38.4)	0.429^¶^
Surgeon experience n (%)	Resident	24 (16.9)	20 (27.4)	17 (25.4)	0.849^ƒ^
	Staff	118 (83.1)	53 (72.6)	50 (74.6)	
Setting n (%)	Elective	120 (84.5)	12 (16.2)	10 (14.7)	0.821^ƒ^
	Emergency	22 (15.5)	62 (83.8)	58 (85.3)	
Outpatient surgery n (%)	No	114 (80.3)	12 (16.2)	16 (23.5)	0.298^ƒ^
	Yes	28 (19.7)	62 (83.8)	52 (76.4)	
Hospital admission time	Days (SD)	1.48 (1.51)	1.58 (1.46)	1.37 (1.57)	0.198^¶^
Resumption of daily life activities/job	Days (SD)	12.4 (16.2)	13.8 (16.3)	10.8 (16.1)	0.314^¶^
Radiological follow up	Days (SD)	670 (290)	743 (312)	586 (240)	**0.005**^**†**^
	Min	223			
	Màx	1294			
Lost to follow up n (%)		27 (18.9)	11 (14.7)	16 (23.5)	0.176^¶^
Primary and secondary outcomes					
TSIHu n (%)		33 (28.2)	16 (25.4)	17 (31.5)	0.538^ƒ^
TSIHu OR (CI)			0.855 (0.568–1.29)		
TSIHc n (%)		18 (17.8)	9 (16.1)	9 (20)	0.613^ƒ^
TSIHc OR (CI)			0.855 (0.568–1.29)		
Surgical site event: n (%)					
Hematoma		3 (2.5)	2 (3)	1 (1.8)	1.000^ƒ^
Seroma		8 (6.6)	7 (10.6)	1 (1.8)	0.070^ƒ^
Wound infection		4 (3.3)	3 (4.4)	1 (1.8)	0.627^ƒ^

*SD* standard deviation, *CI* confidence interval, *DM* diabetes mellitus, *COPD* chronic obstructive pulmonary disease, *BMI* body mass index, *TSIHu* ultrasound diagnosis of trocar site incisional hernia, *TSIHc* clinical diagnosis of trocar site incisional hernia

^a^Diabetes mellitus/age ≥ 70 years, BMI ≥ 30 and/or wound enlargement

^§^Chi Square

^ƒ^f Fisher

^†^t Student

^¶^U Mann–Whitney

**Table 2:** Risk factor distribution

	Intention to treat		p	Per protocol		p
	TSIHu +	TSIHu –		TSIHu +	TSIHu –	
RF average (SD)	1.62 (0.697)	1.57 (0.709)	0.526^¶^	1.61 (0.7)	1.56 (0.7)	0.73

*RF* risk factor, *SD* standard deviation, *TSIHu* + ultrasonographic presence of trocar site incisional hernia, *TSIHu*–: ultrasonographic absence of trocar site incisional hernia

Sixty-four patients in the intervention group and 52 in the control group were analyzed. The groups were homogeneous in almost all demographic characteristics except for the number of smokers, which was higher in the intervention group (15.1 vs 2.9%; p 0.018) (Table 1).

TSIH was clinically diagnosed in nine cases (16.1%) in the intervention group and also in nine patients (20%) in the control group (OR 0.867 [95% CI 0.514–1.46; p = 0.613]). It was detected by ultrasound in 16 patients (25.4%) in the intervention group and in 17 (31.5%) in the control group (OR 0.855 [95% CI 0.568–1.29 p = 0.538) (Table 1).

There were no differences regarding the total number of risk factor presentation, between those patients who presented TSIH diagnosed ultrasonographically and those who not (Table 2).

Regarding surgical site events (surgical wound infection, seroma, or hematoma) no differences were detected between groups (Table 1).

## Discussion

Our results show that, when studied directly and prospectively, the real incidence of TSIH in selected high-risk patients is much higher than previously reported [6, 8, 9, 23], and the overall prevalence of TSIH in the entire cohort (28.2%) also exceeded most of the rates published in the literature [7, 10–13]. Certain specific factors that could explain the detection of this high prevalence. In our view, the most important is the systematic and directed search for TSIH via physical and ultrasound examination in the entire cohort. Similar systematic investigations in which an imaging test was added [15, 16, 23, 24] to the physical examination alone [8–10] achieved a marked increase in the rates of TSIH detection.

The quality of postoperative follow-up is another key factor, as many studies only include cases in which patients consult for notable symptoms or an incarcerated hernia requiring emergency surgery. Some authors recommend that studies of incisional hernia should have a follow-up of at least 24 months (ideally 36 months) [5]. The present study had a mean follow-up of 670 days (almost two years) as measured by performing an abdominal ultrasound. At the time of the project design, a 3-year follow-up was planned for the entire cohort years, and indeed the first patient enrolled presented a follow-up of 1294 days, or 3.5 years; however, the high incidence of TSIH led us to discontinue the study after a mean follow-up of almost two years.

Furthermore, the initial inclusion criteria requiring the presence of at least one of the risk factors for TSIH mentioned above (DM, BMI ≥ 30 kg/m^2^, age ≥ 70 years and/or enlargement of umbilical fascia) meant that the sample was highly selected. The mean of 1.6 risk factors, and so the prevalence of incisional hernia was higher than in other recently published reports [15, 16, 20, 24].

The prevalence of TSIH diagnosed by ultrasound (the reference diagnostic method [21], was lower in the mesh group, although there were no statistically significant differences between groups (25.4 vs 31.5%; p 0.538). In any case, the rate of TSIH in both groups was unacceptably high. Those patients who presented TSIH diagnosed by ultrasound did not present higher number of risk factors.

The study demonstrates that placing a suprafascial mesh in the umbilical trocar position in patients is ineffective in reducing the incidence of TSIH in a selected higher risk population. There are many possible explanations for this lack of efficacy. Firstly, since there are multiple mesh placement options for the treatment of umbilical hernia, the best position remains a matter of debate. Although the current evidence is unclear, the last joint review led by the European Hernia Society and the American Hernia Society issued a “weak recommendation” to place the mesh in a preperitoneal position. The only randomized trial evaluating the role of prophylactic mesh placement in laparoscopic surgery inserted the mesh in an intraperitoneal position and reported a reduction in TSIH in the intervention arm[20]Other authors have described techniques of prophylactic mesh placement in the intraperitoneal position [19, 25, 26] but no clinical trials have been carried out to validate this proposal.

Mesh size may also be an important factor. Although the bridging repair was routinely performed, the mesh overlap could probably be insufficient. According to our protocol, the mesh should cover the aponeurotic defect at least 1 cm around the incision limits. In this study, the caudal limit was defined by the presence of the umbilical root, which was not removed, in order to facilitate the technique but perhaps this detail could imply that the overlap was limited. The size of the mesh should be adapted according to the area of the abdominal wall defect. Historically it has been established that a mesh overlap of 5 cm in all directions would be adequate to minimize recurrence, although this was based on historical theories [27]. The application of mathematical methods such as those proposed by Tulloh et al. [28] could probably optimize the calculation of mesh area to decrease the TSIH rate. Finally, further research is needed to determine whether the mesh fixation requirements should be comparable for prophylactic and therapeutic purposes.

The pneumoperitoneum was created using the Hasson trocar. As its insertion requires an open technique, it was placed supraumbilically in the middle line at a 90º angle. In this way, the incision would not be subject to the background phenomenon, in which the anterior rectus leaflet defect is displaced relative to the posterior rectus leaflet defect. Surprisingly, subjects with a 45ºangled trocar insertion, who would be subjected to the background phenomenon, may still be at a disadvantage compared to subjects with a 90º angled trocar insertion compared (they may have higher rate of TSIH). One reason for this could be that the visualization of the fascia defect for suturing at the end of surgery could be more difficult. But this question still requires further research [29]. Given the Hasson's trocar size of 12 mm, the length of the incision was probably longer (about 18 mm, according to the mathematical model developed by Blinman et al.). [30]. Probably, the insufficient mesh size and its placement in an anatomical site as vulnerable as the umbilical region may have negatively impacted the efficacy of the proposed technique.

As for the secondary objectives of the study, no significant differences were demonstrated in terms of surgical site events such as seroma, hematoma, or wound infection The mean postoperative hospital stay, the substitution index (number of outpatient surgeries) or the resumption of activities of daily living or work were similar. Therefore, we conclude that mesh placement using the described technique is at least safe and does not cause harm to the patient.

Limitations of the study. Most likely, the main limitation of the study is the size of the prophylactic mesh chosen. Considering that the length of the fascial incision was perhaps underestimated, and due to the special characteristics of the umbilical region, the mesh size may not reach sufficient overlap over the fascia and may not be adequate to provide a protective effect. Even though there is not much research on mesh prophylactic purposes, an 18 mm defect might need a 3 cm minimum overlap, according to the European Hernia Society’s recommendations [31]. In addition, in an effort to preserve the abdominal wall's structure as much as possible and to simplify the mesh fixation process after laparoscopic surgery, the study design left the umbilical root in place. This probably implies that, according to current recommendations, further studies should consider disinsertion of the umbilical stalk and placement of a prophylactic mesh with a larger overlap.

Another limitation is that losses to follow-up were more pronounced in the control group. Although the differences were not statistically significant, this could be considered a selection bias.

Among the strengths of the study are the very homogeneous cohort in with no demographic, pathological, intraoperative, or follow up differences between the two groups, and the lower-than-expected rate of loss to follow-up: 18.9%, compared with the initial estimation of 25%.

We conclude that although the suprafascial mesh placement in the umbilical trocar did not cause complications, it is not an effective measure for lowering the TSIH rate in the high-risk population. Future studies should investigate other prophylactic measures (e.g., an intraperitoneal mesh) and other locations in the abdominal wall that are more resistant than the umbilical region for trocar insertion.

## Data Availability

Not applicable.
